# Overview of the Microenvironment of Vasculature in Vascular Tone Regulation

**DOI:** 10.3390/ijms19010120

**Published:** 2018-01-02

**Authors:** Yean Chun Loh, Chu Shan Tan, Yung Sing Ch’ng, Zhao Qin Yeap, Chiew Hoong Ng, Mun Fei Yam

**Affiliations:** Department of Pharmacology, School of Pharmaceutical Sciences, Universiti Sains Malaysia, Minden Gelugor 11800, Penang, Malaysia; lyc14_pha052@student.usm.my (Y.C.L.); tcs14_pha053@student.usm.my (C.S.T.); cys14_pha057@student.usm.my (Y.S.C.); edwardy3ap@gmail.com (Z.Q.Y.); ngchiewhoong@student.usm.my (C.H.N.)

**Keywords:** vascular tone, signaling mechanism pathways, pharmacological tool, endothelium-derived relaxing factors, enzyme-linked receptors, G-protein-coupled receptors, channel-linked receptors, blood vessels

## Abstract

Hypertension is asymptomatic and a well-known “silent killer”, which can cause various concomitant diseases in human population after years of adherence. Although there are varieties of synthetic antihypertensive drugs available in current market, their relatively low efficacies and major application in only single drug therapy, as well as the undesired chronic adverse effects associated, has drawn the attention of worldwide scientists. According to the trend of antihypertensive drug evolution, the antihypertensive drugs used as primary treatment often change from time-to-time with the purpose of achieving the targeted blood pressure range. One of the major concerns that need to be accounted for here is that the signaling mechanism pathways involved in the vasculature during the vascular tone regulation should be clearly understood during the pharmacological research of antihypertensive drugs, either in vitro or in vivo. There are plenty of articles that discussed the signaling mechanism pathways mediated in vascular tone in isolated fragments instead of a whole comprehensive image. Therefore, the present review aims to summarize previous published vasculature-related studies and provide an overall depiction of each pathway including endothelium-derived relaxing factors, G-protein-coupled, enzyme-linked, and channel-linked receptors that occurred in the microenvironment of vasculature with a full schematic diagram on the ways their signals interact. Furthermore, the crucial vasodilative receptors that should be included in the mechanisms of actions study on vasodilatory effects of test compounds were suggested in the present review as well.

## 1. Introduction

Hypertension is a well-known “silent killer” and is one of the major risk factors for causing cardiovascular disease. It is defined as persistently high blood pressure (BP) exerted against the wall of the arteries. Approximately 13% of the human population of the earth suffers from hypertension, and the majority of them are from developing countries. In 2001, there were more than 32.7% of Malaysians aged ≥18 years old, and 43.5% of Malaysian aged ≥30 years old suffering from hypertension [1].

The World Health Organization (WHO, Geneva, Switzerland) has rated hypertension as one of the deadliest causes of premature deaths worldwide due to its asymptomatic behavior which can lead to concomitant diseases after years, such as stroke. This has aroused the attention of worldwide researchers to continue the discovery of new antihypertensive drugs [2]. However, before the vasculature-related research is carried out, the signaling mechanism pathways happening in the vasculature should be clearly understood. Previous reviews have shown that aortic ring assay is recommended as the “golden tool” in most of the in vitro pharmacological research, especially those related to vasculature, along with the protocols and suggested antagonists that are to be used for different mechanism studies [3,4,5,6].

**Figure 1:**
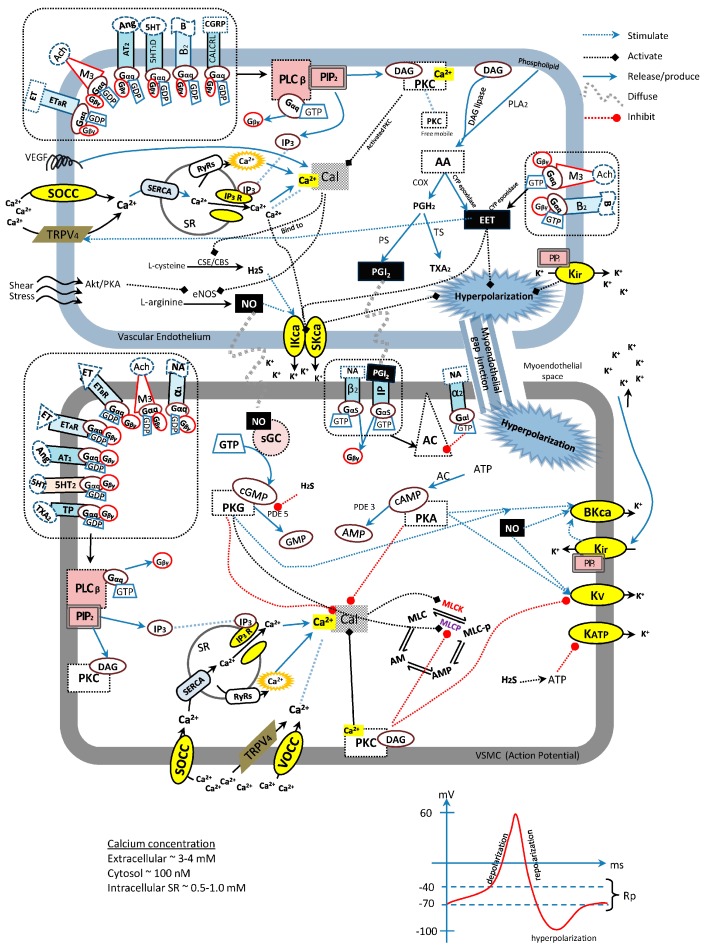
The overview of signaling transduction among endothelium-derived relaxing factors, G-protein-coupled, enzyme-linked, and channel-linked receptors in vascular endothelium and vascular smooth muscle cells during vascular tone regulation.

Despite the fact that there are many articles that have discussed the different signaling mechanism pathways in vasculature, either thoroughly or briefly, none of them explained all of them in full. Hence, the main objective of the present review is to provide an overview regarding the interaction between endothelium-derived relaxing factors, G-protein-coupled, enzyme-linked and channel-linked receptors in vasculature including the activation of different receptors, production of second messengers, and the transduction of signals. All these signals were compiled as a whole (Figure 1) which is essential and serves as a reference for those who are involved in antihypertensive drug research as well as to serve as a guide for any other vasculature-related pharmacological researches. Besides this, types of vasodilative receptors that were most frequently investigated in the study of mechanisms of actions were also suggested.

## 2. Blood Vessel

There are three types of blood vessels–the artery, vein, and capillary—responsible for transporting blood throughout the whole body. There are three layers present in artery and vein, where the outermost layer is known as tunica adventitia, the middle layer as tunica media, and the innermost layer as tunica intima. Tunica media is rich in vascular smooth muscles (VSMCs), whereas tunica intima is composed of a thin layer of endothelial cells. Both the VSMCs and endothelium are the place where the vasomotors are located [7,8]. The vasomotors can cause the vascular tone to react in two ways such as vasodilation or vasoconstriction where both reactions are strictly dependent on the dominancy of the receptors present on the vascular endothelium and VSMCs as well as the interactions between their signals.

## 3. Endothelium-Derived Relaxing Factors (EDRFs)

There are two well-characterized EDRFs present in endothelium: nitric oxide (NO) and prostacyclin (PGI_2_). Additionally, the endothelium-derived hyperpolarizing factor (EDHF), and hydrogen sulfide (H_2_S) have been claimed to be one of the EDRFs as well recently.

### 3.1. Nitric Oxide

Generally, the NO can be produced by three isoforms of NO synthase (NOS)—neuronal NOS (nNOS), inducible NOS (iNOS), and endothelial NOS (eNOS). In endothelium, the increase in the concentration of the calcium in cytosol will enhance the formation of calcium–calmodulin complexes, which will activate the calmodulin-binding domain of the eNOS, and causes NO production. Other than that, the increase in hemodynamic shear stress, protein kinase A (PKA), and protein kinase B (Akt) in the blood vessel will induce the phosphorylation of the eNOS at Ser1179 site [9,10]. Once the eNOS is activated, it will catalyze the breakdown of l-arginine into NO. Subsequently, NO will diffuse into adjacent VSMCs to stimulate the activity of the components down its signaling cascade such as soluble guanylyl cyclase (sGC), cyclic guanosine monophosphate (cGMP), and protein kinase G (PKG), hence resulting in vasodilation [7,11]. The production of the NO can further stimulate the K_ca_ channels [12,13] and voltage-activated K^+^ channels (K_v_) through the sGC-independent pathway [14]. Regarding the mechanism study, the selective antagonist of eNOS, L-N^G^-nitroarginine methyl ester (L-NAME) is frequently used due to its low toxicity, and more solubility at neutral pH compared to other inhibitors [15,16].

### 3.2. Prostacyclin (PGI_2_)

This is also known as prostaglandin I2, and is well-known as one of the important EDRFs which is capable of inhibiting platelet aggregation. In endothelium, the precursor of the PGI_2_ is arachidonic acid (AA) which ordinarily exists in the phospholipid bilayer of membranes. The AA will be released into the cytosol either catalyzed by phospholipase A_2_(PLA_2_) or diacylglycerol (DAG) lipase by breaking down the phospholipid. The free mobile AA will be converted into prostaglandin H_2_ (PGH_2_) by cyclooxygenase (COX). Subsequently, the prostacyclin synthase will catalyze the breakdown of PGH_2_ into PGI_2_, whilst some of the PGH_2_ is converted into thromboxane (TXA_2_) by thromboxane synthase. Both the TXA_2_ and PGI_2_ functions are physiologically antagonists. The PGI_2_ will bind to the prostacyclin receptor (IP) which is located on the membrane of the VSMCs. IP is a G_s_α-protein-coupled receptor, once the G_s_α-protein is bound to guanosine triphosphate (GTP), the membrane-bound adenylyl cyclase (AC) will be stimulated to convert the adenosine triphosphate (ATP) into 3′,5′-cyclic adenosine monophosphate (cAMP), which will then activate the PKA, and causes vasodilatory effects [17,18]. In the mechanism studies, the non-selective COX inhibitor, indomethacin which is a non-steroidal anti-inflammatory drug (NSAID) is frequently used compared to others such as ibuprofen, meclofenamic acid, and diclofenac. Indomethacin is preferred because it can bind rapidly to the COX with high-affinity, is time-dependent and has slow reversibility [19,20].

### 3.3. Endothelium-Derived Hyperpolarizing Factors

EDHFs were discovered when there were residuals of endothelium-dependent relaxation observed even after the depletion of both NO and PGI_2_ [21,22]. It is a kind of electrical signal that originated from the endothelium, which could subsequently induce the hyperpolarizing current in adjacent VSMC and cause a vasodilatory effect. In vasculature, EDHF can be generated through various kinds of reactions that happen between different biochemical components. For instance, part of the AA in the endothelium could be broken down into epoxyeicosatrienoic acids (EETs) by cytochrome P450 (CYP) epoxygenase, at which the EETs could activate the small-conductance Ca^2+^-activated K^+^ channels (SK_ca_) and intermediate-conductance Ca^2+^-activated K^+^ channels (IK_ca_) which is located on the endothelium, hence causing the K^+^ ions efflux and inducing the hyperpolarizing current that will be passed onto the adjacent VSMCs via the myoendothelial gap junction, which was electrically-coupled between both endothelial cells and VSMCs, ultimately causing the VSMCs’ hyperpolarization and relaxation. Furthermore, the EETs can activate the transient receptor potential vanilloid 4 (TRPV4) that is located on the endothelium to allow Ca^2+^ influx into the cytosol, and subsequently causes an increase of intracellular calcium concentration (Ca^2+^ spark) due to the calcium-induced calcium release (CICR) reaction, when the influx cytosolic Ca^2+^ stimulates the ryanodine receptors (RyRs) that are located on the sarcoplasmic reticulum (SR). However, in terms of action potential, the efflux of K^+^ ions into the myoendothelial space leads to an increase in concentration of K^+^, causing the activation of the inwardly-rectifying K^+^ channels (K_ir_) on the VSMCs to allow the influx of K^+^ ions back into the cytosol of VSMCs, and followed by efflux of K^+^ ions through the big-conductance Ca^2+^-activated K^+^ channels (BK_ca_) to extracellular and causes hyperpolarization, resulting in vasodilatory effect [23,24].

### 3.4. Hydrogen Sulfide

H_2_S has a similar chemical profile as NO in vasculature. In the endothelium, the H_2_S is produced from the l-cysteine which is catalyzed by cystathionine γ-lyase (CSE) and/or cystathionine β-synthase (CBS). The H_2_S is capable of activating the IK_ca_ and SK_ca_ channels in endothelium, hence producing the hyperpolarizing current. The literature has stated that the production of H_2_S could be increased at least two-fold in endothelium when triggered by the VEGF and muscarinic receptor activation through the Ca^2+^-calmodulin-dependent activation of the CSE [25,26]. Moreover, H_2_S is diffusible into the adjacent VSMCs to inhibit the ATP from binding with ATP-sensitive K^+^ channels (K_ATP_), hence activating the channels and subsequently allowing the K^+^ efflux which results in hyperpolarization. H_2_S is also capable of inhibiting the phosphodiesterase 5 (PDE5) from breaking down the cGMP, hence enhances the vasodilatory effects.

## 4. Enzyme-Linked Receptors

These receptors can be called catalytic receptors and are located on the membrane. These receptors are functionally activated by catalytic enzymes and ligand-receptors. In vasculature, the guanylyl cyclase and serine-threonine protein kinases are the major enzyme-linked receptors that play the roles in vascular tone regulation.

### 4.1. Soluble Guanylyl Cyclase

As aforementioned, the sGC is freely mobile in the cytosol of VSMCs which will be activated once the NO comes to bind with the heme group of sGC. Once the sGC is activated, it will cleave the GTP into cGMP, subsequently activates the PKG, and causes vasodilatory effect [27,28]. The 1H-[1,2,4] oxadiazolo [4,3-a] quinoxalin-1-one (ODQ) is soluble in dimethyl sulfoxide (DMSO) and was reported to inhibit the sGC by oxidizing the heme group of the sGC [29,30,31] and it is more frequently used compared to the other cGMP lowering agent, methylene blue (MB) [32,33].

### 4.2. Serine-Threonine Protein Kinases

This is basically a group of kinase enzymes that is functionally activated when bound to their respective second messenger, and subsequently phosphorylates the hydroxyl (OH) group of serine or threonine side chain of proteins which results in physiological effects. In vascular tone regulation, the protein kinases involved are PKA, protein kinase C (PKC), and PKG.

PKA is also known as the cAMP-dependent protein kinase, which is functionally activated once bound with cAMP. Once bound with cAMP, its detaching catalytic subunits will phosphorylates the serine or threonine sites of its substrate proteins, and causes vasodilatory effect, whilst the cAMP will be broken down into adenosine monophosphate by phosphodiesterase 3 (PDE3) [34]. Regarding this mechanism study on cAMP-dependent PKA pathway, the Rp-cAMPs is commonly used due to its cell-permeability, resistance to PDE degradation as well as its selectivity towards PKA [35]. Additionally, the selective inhibitor for PDE 3 that is commonly used is milrinone.

In both vascular endothelium and VSMCs, the PKC will be activated by DAG and binds with Ca^2+^ ions at C1 and C2 domain, respectively [36]. Once the PKC is activated, it will phosphorylate the serine or threonine sites of its substrate proteins, hence causes vasoconstriction. The most commonly used blocker for PKC is BIM due to its high permeability and selectivity.

As aforementioned, the PKG will be functionally activated once the cGMP binds to the regulatory units of the PKG without causing enzyme dissociation. After that, the activated catalytic units of the enzyme will phosphorylates the serine or threonine sites of its substrate proteins, and causes vasodilatory effect [37], whilst the cGMP will be broken down into guanosine monophosphate (GMP) which is catalyzed by PDE 5 [38]. The most commonly used cGMP-dependent PKG inhibitor is Rp-8-Br-PET cGMPs due to its high stability and the ability to block both PKG 1 and PKG 2 [39]. Additionally, the selective inhibitors for PDE 5 commonly used are sildenafil, dipyridamole, zaprinast and T-1032.

## 5. G-Protein-Coupled Receptors (GPCRs)

GPCRs are also known as the seven-transmembrane domain receptors, which are functionally activated when bound to its ligand to transmit the signals from the outside of the cell into its interior. Generally, there are three types of guanine nucleotide binding protein (G-protein) subunits–Gα-, Gβ-, and Gγ-proteins—and the former is the one which plays the major role in vascular tone regulation and is further classified into at least three main sub-types—G_q_α, G_i_α, and G_s_α. The activation of the G-protein will be initiated once the GPCRs bind to its ligand which subsequently causes the G-protein to temporarily act as guanine nucleotide exchange factor (GEF) by changing its conformation, and exchange its guanosine diphosphate (GDP) into GTP. Once bound with GTP, the G-protein trimer will be dissociated into Gα-GTP monomer and Gβγ dimer. Gα-GTP monomer will start to interact with their intracellular proteins for signal transduction and will be discussed in below section [40], whereas Gβγ dimer will tend to activate certain types of signaling molecules including ion channels, lipid kinases, phospholipases as well as its own signaling cascades [41,42].

### 5.1. G_q_α-Protein-Coupled Receptors

In vasculature, the activation of the G_q_α-protein will cleave the phosphatidylinositol 4,5-bisphosphate (PIP_2_) into second messengers, inositol triphosphate (IP_3_) and DAG by binding its G_q_α-subunit to the phospholipase C (PLC). The IP_3_ is soluble and diffusible in cell and binds to the intracellular receptor, IP_3_ receptor (IP_3_R) which is located inside the cell and on the SR to trigger the intracellular release of the Ca^2+^ ions from the SR into the cytosol. Whereas, the DAG will activate the PKC as described above and result in the increase of Ca^2+^ concentration in cytosol. In vasculature, the G_q_α-protein-coupled receptors that are present in endothelium includes angiotensin-2 receptor (AT_2_), serotonin receptor (5-HT_1D_), bradykinin receptor (B_2_), muscarinic-3 receptor (M_3_), endothelin-B receptor (ET_B_R), and calcitonin receptor-like receptor (CALCRL), whereas there are α_1_-adrenergic receptor (α_1_), M_3_-muscarinic receptor, angiotensin-1 receptor (AT_1_), endothelin receptors (ET_A_R & ET_B_R), serotonin receptor (5-HT_2_), and TXA_2_ receptor present in VSMCs [7,8,43,44,45,46,47,48].

### 5.2. G_i_α-Protein-Coupled Receptors

In vasculature, there is a G_i_α-protein-coupled receptor present in the VSMCs such as α_2_-adrenergic receptor (α_2_). Once this receptor is activated by its agonist, it will inhibit the activity of the cAMP-dependent AC to convert ATP into cAMP, hence causes vasoconstriction [48,49].

### 5.3. G_s_α-Protein-Coupled Receptors

Typically, the G_s_α-protein-coupled receptor is functionally opposed to G_i_α-protein-coupled receptor, where it will activate AC to produce cAMP from ATP, the increase in the production of cAMP will subsequently enhance the activation of PKA as mentioned above and result in a vasodilatory effect. There are at least two major G_s_α-protein-coupled receptors present in VSMCs such as β_2_-adrenergic receptor (β_2_) and PGI_2_ receptor (IP) [7,48]. In addition, the commonly used AC inhibitor for mechanism study is SQ22536 because it is more selective towards AC, higher cell-permeability and better solubility.

## 6. Channel-Linked Receptors

These channels are also known as ion channel-linked receptors and/or ligand-gated receptors and/or ionotropic receptors. These receptors will be functionally activated when bound to its ligand and allowing ions such as sodium (Na^+^), potassium (K^+^), chloride (Cl^−^), and calcium (Ca^2+^) move through the membrane. In vasculature, the action potential which occurs in VSMCs will be regulated by these receptors through hyperpolarization or depolarization. There are two important channel-linked receptors playing major roles in vascular tone regulation which are K^+^ and Ca^2+^ channels.

### 6.1. Potassium Channels

K^+^ channel is the most widely distributed type of ion channel in living organisms [50]. Typically, there are four types of K^+^ channels frequently discussed and investigated in vascular tone regulation which includes calcium-activated K^+^ channel (K_ca_), voltage-gated K^+^ channel (K_v_), ATP-sensitive K^+^ channel (K_ATP_), and inwardly-rectifying K^+^ channel (K_ir_).

In human vasculature, K_ca_ channel can be further divided into three subtypes: big-conductance K_ca_ channel (BK_ca_), intermediate-conductance K_ca_ channel (IK_ca_), and small-conductance K_ca_ channel (SK_ca_). BK_ca_ channel is widely distributed in VSMCs, whereas IK_ca_ and SK_ca_ channels are abundantly expressed in endothelium [7,51,52,53]. The electric conductance for BK_ca_, IK_ca_, and SK_ca_ channels ranges between 100–300, 25–100, and 2–25 Picosiemens (pS), respectively. In VSMCs, the BK_ca_ channel is voltage and Ca^2+^-dependent, and it will be activated by the increased intracellular Ca^2+^ concentration, which allows the efflux of K^+^ ions from the cytosol, hence creating hyperpolarization and the closure of the Ca^2+^ channels, which results in vasodilatory effect [54,55]. Furthermore, BK_ca_ channel can be indirectly stimulated by the activation of PKA and PKG [56,57]. IK_ca_ and SK_ca_ channels are slightly different from BK_ca_ channel by which they are voltage-insensitive [58,59,60], but highly sensitive to the Ca^2+^ and calmodulin concentration in cytosol [61,62]. The most commonly used selective antagonists for BK_ca_, IK_ca_, and SK_ca_ are iberiotoxin, clotrimazole, and apamin, respectively.

In vasculature, the activation of K_v_ channel is strictly dependent on the voltage changes across the membrane and is functionally correlated with the voltage-operated Ca^2+^ channel (VOCC) in maintaining the membrane potential of VSMCs. K_v_ channel will reverse the depolarizing state ofthe membrane potential back to steady state [63]. Typically, there are two major subunits for K_v_ channel, which are the alpha subunits (can be further grouped into 12 subclasses), and the beta subunits. They are functionally activated to hasten the efflux of K^+^ from the cytosol out to the exterior, hence a more rapid increase of repolarizing current, with less activation of Ca^2+^ channels [7,8,64]. In addition, the cAMP-dependent PKA can indirectly increase the amplitude of the K_v_ currents [65] and inhibited by the activation of PKC [66]. The commonly used inhibitor for its mechanism study is 4-aminopyridine (4-AP).

This channel contains a pore domain, which is homologous to K_v_ channel, and therefore it has been classified as one of the members in the K_v_ channel. The K_ir_ channel needs to bind with PIP_2_ to be activated, and functionally prefers the inward flow of the K^+^ ions rather than outward, hence hastening the recovery of the membrane potential back to resting state [54,67,68]. The activation of this channel in endothelium will contribute as EDHFs for inducing the relaxing of VSMCs [69]. The only difference of K_ir_ channel compared to other K^+^ channels is it will only be activated when the membrane potential has reached hyperpolarization state, then allowing the influx of K^+^ ions into the cytosol to reach resting potential [70]. The only selective antagonist used for the K_ir_ channel study is barium chloride (BaCl_2_).

This channel will be activated by increasing intracellular ADP and decreasing intracellular ATP, also called ATP-sensitive channel and is located in VSMCs [28,71,72,73]. In resting potential, K_ATP_ channel acts as a weak inwardly rectifying K^+^ channel, thus it was classified as a member in K_ir_ channel family. However, once K_ATP_ channel is activated, it will produce K^+^ efflux from the cytosol to maintain a negative resting potential, hence causing vasodilatory effect [7]. The most commonly used selective K_ATP_ channel inhibitor for mechanism study is glibenclamide.

### 6.2. Calcium Channels

The Ca^2+^ ion-linked receptor is selectively permeable for Ca^2+^ ions and allows its entrance into the cytosol, hence creating depolarization and inducing vasoconstriction. Typically, there are two different types of Ca^2+^ channel: VOCC and receptor-operated Ca^2+^ channels (ROCC). Generally, there are two ways to increase the cytosolic Ca^2+^ concentration which are through (1) the influx of Ca^2+^ ions from exterior or the (2) intracellular release of Ca^2+^ from the SR store. Ca^2+^ ion is one of the most crucial second messengers in vascular tone regulation. The intracellular increase of Ca^2+^ concentration will cause membrane depolarization and allow the up-regulation of Ca^2+^-calmodulin complexes. In the cross-bridge cycle in VSMCs, the activated calmodulin will stimulate the MLC kinases (MLCK) to phosphorylate the MLC at serine residue-19 to form a cross-bridge with the actin filament, and again form actin-myosin protein (AMP), hence causes the VSMCs contraction via the sliding filament mechanism [7,53,74,75]. In addition, there is another enzyme in this cross-bridge, the MLC phosphatases (MLCP) which can dephosphorylate the MLC in order to terminate the smooth muscle contraction.

In vasculature, VOCC is one of the most important receptors used to control the vascular tone by maintaining the membrane potential of the VSMCs. Here, the VOCC is normally referred to as the L-type Ca^2+^ channel that is in VSMCs. In normal physiological condition, the concentration of the Ca^2+^ ions outside the cell is around 3–4 mM which is a thousand-fold higher than inside the cell, which is normally kept at or below 100nM [76]. Therefore, once the membrane potential reached depolarization state, the Ca^2+^ ions will rush into the cytosol from the outside via VOCC, hence results in vasoconstriction [46]. It is functionally correlated with K_v_ channel in controlling the membrane potential. The commonly used antagonist for this channel mechanism study is nifedipine due to its highly vascular selectivity [77].

The calcium cannot only enter the VSMCs by Ca^2+^ influx via VOCC, but also through intracellular Ca^2+^ release via the ROCC that could lead to the membrane depolarization [78,79]. The ROCC normally refer to certain members of GPCRs that are capable of inducing the intracellular release of Ca^2+^ ions from the SR store into the cytosol by producing its second messenger [76,80]. Typically, there are at least three types of receptors that are categorized as ROCC such as IP_3_R, RyRs, and store-operated Ca^2+^ channels (SOCC).

The IP_3_R is located on the surface of the SR, which will be activated by the second messenger, IP_3_, that is produced by activated G_q_α-protein-coupled receptors as described above. It is the main site for the intracellular Ca^2+^ release from the SR store into the cytosol which leads to an increase in the formation of Ca^2+^-calmodulin complexes [81]. Regarding to this mechanism study, the selective IP_3_R blocker, 2-aminoehtoxydiphenyl borate (2-APB) is commonly used.

This receptor is less likely distributed in vascular, however, it plays minor role in controlling the intracellular release of Ca^2+^ ions into the cytosol by using calcium-induced calcium release (CICR). The increasing Ca^2+^ ions concentration in cytosol will trigger the RyRs to release more Ca^2+^ ions from the SR store which subsequently causes a transient Ca^2+^ spark, which is important for muscle contraction.

The primary function of SOCC is to refill Ca^2+^ ions, and it is known as a capacitative-dependent calcium entry channel, whereby its activation across the plasma membrane will occur with the depletion of the Ca^2+^ stores [54,76]. The sacro/endoplasmic reticulum Ca^2+^-ATPase (SERCA) is the only specialized pump used to transport the Ca^2+^ ions from the extracellular into the SR store to re-accumulate the calcium concentration back to the range between 0.5–1 mM. In the SR store, the Ca^2+^ ions will bind to calsequestrin with the purpose of decreasing the free mobile Ca^2+^ ions in the SR, hence more calcium can be stored [82,83,84]. The selective SOCC blocker, gadolinium (Gd^3+^) is usually accompanied with the use of the selective blocker of SERCA, thapsigargin [85] during the mechanism study of SOCC pathway.

## 7. General Integration of Vasodilative Receptors

Generally, there are five crucial types of second messenger that could directly affect the vascular tone: DAG, IP_3_, cGMP, cAMP, and Ca^2+^ ions. The production of these second messengers are strictly dependent on the activation of various types of the receptors that are located on the vasculature including EDRFs, enzyme-linked, G-protein-coupled, and channel-linked receptors. According to literatures, the vasodilative receptors are more frequently being included in the experimental study on mechanisms of actions of test compounds rather than vasoconstrictive receptors [86,87,88,89,90,91]. Therefore, following the current trend of pharmacological research, all the vasodilation-mediated receptors pathways that should be investigated during the mechanisms of actions study of test compounds were suggested at this section.

In vascular endothelium, the eNOS and COX that are mainly responsible as the catalyst for the production of two well-known EDRFs, such as NO and PGI_2_, respectively, should be investigated. The increase of NO in the vascular endothelium could activate the IK_ca_ and SK_ca_, and results in the efflux of K^+^ ions into the myoendothelial space. The activation of the potassium channels in the vascular endothelium would create a hyperpolarizing current, known as EDHF, which would be transmitted through the myoendothelial gap junction to the adjacent VSMCs and cause vasodilation. Besides this, NO can diffuse through the adjacent VSMCs to activate the sGC for the production of cGMP and result in vasodilation. Furthermore, PGI_2_ produced by COX in the vascular endothelium will bind to the IP receptor that located on the membrane of the adjacent VSMCs, and causes the activation of AC and up-regulation of cAMP; hence, this results in vasodilation. Therefore, NO/sGC/cGMP and COX are major pathways that should be investigated during the mechanisms of actions study on vasodilatory effects of test compounds [5,86].

Besides the activation of IP receptor, the G_s_α-protein-coupled β_2_-adrenergic receptors that are located on the VSMCs also possess a similar mechanism event, where its activation will cause a vasodilatory effect when bound to its agonist. There is another G_q_α-protein-coupled receptor that is located in both vascular endothelium and VSMCs, but functionally predominant in vascular endothelium, which is the M_3_ receptor. When the M_3_ receptor binds to its agonist, the PLCβ will be activated to produce IP_3_ and DAG. At this stage, IP_3_ will bind to the IP_3_R that is located on the membrane of the sacroplasmic reticulum to allow the Ca^2+^ ions efflux from the intracellular to the cytosol, hence increasing the formation of Ca^2+^-calmodulin complexes, whereas DAG will bind and activate PKC, which increases the formation of Ca^2+^-calmodulin complexes. The increasing production of the Ca^2+^-calmodulin complexes in the vascular endothelium will activate both the eNOS and CSE to produce NO and H_2_S, respectively, and resulting in vasodilation. Therefore, both G_s_α-protein-coupled β_2_-adrenergic and G_q_α-protein-coupled M_3_ receptors pathways are essential to be included in the mechanisms of actions study on vasodilatory effects of test compounds [5,90,91].

According to Figure 1, there are four major potassium channels, which are K_ca_, K_v_, K_ir_, and K_ATP_ channels, and the calcium channel, VOCC, which are playing crucial roles in regulating the membrane potential of the VSMCs. The activation of potassium channels will cause the efflux of the K^+^ ions out from the cytosol, and creates a repolarizing/hyperpolarizing current on the membrane of the VSMCs. During this stage, the VOCC will be closed, hence causing a vasodilation. Subsequently, the slow influx of the K^+^ ions through the K_ir_ channel will cause the membrane potential to reach resting state. After the resting state, the depolarizing current will start to increase via the activation of VOCC, allowing the entrance of Ca^2+^ ions from the extracellular into the cytosol, and causing the increasing formation of Ca^2+^-calmodulin complexes. The increasing formation of the Ca^2+^-calmodulin complexes in VSMCs will promote the forward reactions of the MLC cross-bridge, which will cause vasoconstriction. Other than VOCC, IP_3_R has played a major role in vascular tone regulation as well. The activation of IP_3_R is strictly dependent on the activation of G_q_α-protein-coupled receptors, which allows the Ca^2+^ ions released from the store of sacroplasmic reticulum into the cytosol. Therefore, all these channel-linked pathways should be investigated during the mechanisms of actions study on vasodilatory effects of test compounds [5,86,87,88,89,90,91].

## 8. Conclusions

The overall interaction between different EDRFs, G-protein-coupled, enzyme-linked, and channel-linked receptors as well as their second messengers including cGMP, cAMP, IP_3_, DAG, and Ca^2+^ in vascular endothelium and VSMCs during vascular tone regulation has been successfully compiled as a whole. This could serve as a useful tool for any future antihypertensive drug research, and also may serve as a guide for other vasculature-related pharmacological research. Furthermore, the NO/sGC/cGMP, PGI_2_, G_q_α-protein-coupled M_3_-muscarinic and G_s_α-protein-coupled β_2_-adrenergic receptors, K_ca_, K_v_, K_ir_, K_ATP_, VOCC, and IP_3_R pathways which are crucial in causing vasodilation were suggested to be included in the experimental study on mechanisms of actions of test compounds.

## Abbreviations

α	α-adrenergic receptor
β	β-adrenergic receptor
2-APB	2-Aminoethoxydiphenyl borate
4-AP	4-Aminopyridine
5-HT	5-Hydroxytryptamine
AA	Arachidonic acid
AC	Adenylyl cyclase
AHA	American Heart Association
AMP	Adenosine monophosphate
Akt	Protein kinase B
ATP	Adenosine triphosphate
AT	Angiotensin receptor
B_2_	Bradykinin receptor B_2_
BaCl_2_	Barium Chloride
BK_ca_	Big conductance calcium-activated potassium channel
BP	Blood pressure
CALCRL	Calcitonin receptor-like
cAMP	Cyclic adenosine monophosphate
CBS	Cystathionine β-synthase
cGMP	Cyclic guanosine monophosphate
CICR	Calcium-induced calcium release
COX	Cyclooxygenase
CSE	Cystathionine γ-lyase
CYP	Cytochrome P450
DAG	Diacylglycerol
DMSO	Dimethyl sulfoxide
eNOS	Endothelium nitric oxide synthase
EDHF	Endothelium-derived hyperpolarizing factor
EDRFs	Endothelium-derived relaxing factors
EETs	Epoxyeicosatrienoic acids
ETR	Endothelin receptor
Gd^3+^	Gadolinium
GDP	Guanosine diphosphate
GEF	Guanine nucleotide exchange factor
GMP	Guanosine monophosphate
GPCRs	G protein-coupled receptors
GTP	Guanosine triphosphate
H_2_S	Hydrogen sulfide
IK_ca_	Intermediate conductance calcium-activated potassium channel
IP	Prostacyclin receptor
IP_3_	Inositol triphosphate
iNOS	Inducible nitric oxide synthase
JNC7	Seventh report of Joint National Committee
K_ATP_	ATP-sensitive potassium channel
Kca	Calcium-activated potassium channel
K_ir_	Inwardly-rectifying potassium channel
K_v_	Voltage-activated potassium channel
L-NAME	L-N^G^-Nitroarginine methyl ester
M_3_	Muscarinic receptor M_3_
MB	Methylene blue
MLCK	Myosin light-chain kinase
MLCP	Myosin light-chain phosphatase
NO	Nitric oxide
NOS	Nitric oxide synthase
nNOS	Neuronal nitric oxide synthase
NSAIDs	Non-steroidal anti-inflammatory drugs
ODQ	1H-[1,2,4] oxadiazolo [4,3,-a] quinoxalin-1-one
PDE3	Phosphodiesterase 3
PDE5	Phosphodiesterase 5
PGH_2_	Prostaglandin H2
PGI_2_	Prostacyclin
PIP_2_	Phosphatidylinositol 4,5-bisphosphate
PKA	Protein kinase A
PKG	Protein kinase G
PLA_2_	Phospholipase A_2_
PLC	Phospholipase C
ROCC	Receptor-operated calcium channel
RyRs	Ryanodine receptors
SERCA	Sacro/endoplasmic reticulum Ca^2+^-ATPase
sGC	Soluble guanylyl cyclase
SK_ca_	Small conductance calcium-activated potassium channel
SOCC	Store-operated calcium channel
SR	Sacroplasmic reticulum
TRPV_4_	Transient receptor potential vanilloid 4
TxA_2_	Thromboxane
VOCC	Voltage-operated calcium channel
VSMCs	Vascular smooth muscle cells
WHO	World Health Organization
